# [Bis(2-pyrid­yl)amine-*N*,*N*′](nitrato-*O*,*O*′)cobalt(II) nitrate. Corrigendum

**DOI:** 10.1107/S1600536810050798

**Published:** 2011-02-12

**Authors:** Oscar Castillo, Antonio Luque, Noelia De la Pinta, Pascual Román

**Affiliations:** a Departamento de Química Inorgánica, Facultad de Ciencias, Universidad del País Vasco, Apdo. 644, E-48080 Bilbao, Spain; b Departamento de Química Inorgánica, Facultad de Ciencias, Universidad del País Vasco, Apdo. 644, E-48080 Bilbao, Spain

## Abstract

The chemical name and formula in the paper by Castillo, Luque, De la Pinta & Román [*Acta Cryst.* (2001), E**57**, m384–m386] is corrected.

## Abstract 2

Corrigendum to *Acta Cryst.* (2001), E**57**, m384–m386.

In the paper by Castillo, Luque, De la Pinta & Román (2001 ▶), the ligand reported as nitrate should be carbonate and the oxidation state of the cobalt metal atom should be Co^III^ rather than Co^II^, thus making the correct chemical composition [Co(CO_3_)(C_10_H_9_N_3_)_2_]NO_3_ and the correct chemical name ‘[Bis(2-pyri­dyl)amine-κ^2^ *N*,*N*′](carbonato-κ^2^ *O*,*O*′)cobalt(III) nitrate’.

## Experimental

### 

#### Crystal data

[Co(CO_3_)(C_10_H_9_N_3_)_2_]NO_3_
*M* *_r_* = 523.35
Monoclinic, 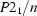

*a* = 17.191 (3) Å
*b* = 7.3080 (10) Å
*c* = 17.843 (5) Å
β = 104.94 (3)°
*V* = 2165.9 (8) Å^3^
*Z* = 4
Mo *K*α radiation
μ = 0.85 mm^−1^
*T* = 293 K
0.42 × 0.20 × 0.08 mm

#### Data collection

Stoe IPDS diffractometer
Absorption correction: numerical (Stoe & Cie, 1998 ▶) *T*_min_ = 0.815, *T*_max_ = 0.934
14084 measured reflections
4037 independent reflections
2598 reflections with *I* > 2σ(*I*)
*R*_int_ = 0.048

#### Refinement

*R*[*F* ^2^ > 2σ(*F* ^2^)] = 0.031
*wR*(*F* ^2^) = 0.076
*S* = 0.82
4037 reflections
316 parameters
H-atom parameters constrained
Δρ_max_ = 0.37 e Å^−3^
Δρ_min_ = −0.31 e Å^−3^

### 

Data collection, cell refinement and data reduction: *IPDS Software* (Stoe & Cie, 1998 ▶); program(s) used to solve structure: *SIR92* (Altomare *et al.*, 1993 ▶); program(s) used to refine structure: *SHELXL93* (Sheldrick, 1993 ▶).

## Supplementary Material

Crystal structure: contains datablocks global, I. DOI: 10.1107/S1600536810050798/bt9068sup1.cif

Structure factors: contains datablocks I. DOI: 10.1107/S1600536810050798/bt9068Isup2.hkl

. DOI: 10.1107/S1600536810050798/bt9068fig1.tif
?

Additional supplementary materials:  crystallographic information; 3D view; checkCIF report
